# Inspiring sustainability: insights from a global creative competition on healthy planet, healthy people

**DOI:** 10.1177/17579139241249676

**Published:** 2024-11-06

**Authors:** Stephanie Bull, Elen Evans, Jonas Nzanzu, Fadhilah Ramadhina, Jamie Thompson, Thomas Cooper, Tracy Moniz, Alice O’Grady, Ravi Parekh, Tom Rozier-Hope, Sukhi Ubhi, Sonia Kumar

**Affiliations:** Medical Education Innovation and Research Centre (MEdIC), Imperial College London, London W127TA, UK; Cardiff University, Cardiff, UK; Université Simon Kimbangu, Kinshasa, Democratic Republic of the Congo; Universitas Indonesia, Depok, Indonesia; Northern Ontario School of Medicine University, Calgary, AB, Canada; University of Leeds, Leeds, UK; Department of Communication Studies, Mount Saint Vincent University, Halifax, NS, Canada; University of Leeds, Leeds, UK; Medical Education Innovation and Research Centre (MEdIC), Imperial College London, London, UK; Medical Education Innovation and Research Centre (MEdIC), Imperial College London, London, UK; Medical Education Innovation and Research Centre (MEdIC), Imperial College London, London, UK; University of Leeds, Leeds, UK

*In this article, Bull et al. focus on the Global Creative Competition and how it allowed medical students from across the world to reflect on the welfare of people and our planet. It provided an opportunity for students from different healthcare disciplines and across low- and high-income countries to come together to discuss their commitment to transformational change*.

## Introduction

The United Nations’ 17 sustainable development goals (SDGs) aim to address poverty, improve health and education, and reduce inequality alongside tackling climate change by 2030.^1^ The impact of these interrelated SDGs, whether direct or indirect – on people’s health and wellbeing, was highlighted as a priority at the 28th session of the conference of the parties (COP28),^2^ particularly the interdependency between health and climate.

Climate disruption directly undermines health by increasing extreme weather events and wildfires, reducing food security, and spreading infectious diseases.^3^ Healthcare is also a major contributor of carbon emissions. If the global healthcare system were a country, it would be the fifth largest carbon emitter.^4^ It is, therefore, essential that healthcare professionals, present and future, across the globe engage with each other in shaping how healthcare can be delivered sustainably.^5^ Engagement in the arts is an effective tool for promoting reflective practice in health professions education. A key role of the arts in this context is to encourage social advocacy, including seeking transformative changes in healthcare and in society at large.^6^

## Methods

The Global Creative Competition was established in 2020 for medical students to understand and reflect on the global COVID-19 pandemic.^7^ Over the past four years, the competition has developed and in 2023, it evolved into a portal for healthcare students worldwide to reflect and respond to the SDGs in imaginative ways. Students were asked to produce creative work (e.g. drawing, painting, photography, video, music, poetry) related to the goals and to submit a reflection about what their work meant to them personally and professionally.

## Results

Over 100 entries were received from students on healthcare profession programmes, including medicine, nursing, physiotherapy, dentistry, optometry, and pharmacy, from 17 countries. The number of articles from each country is in parentheses: India (73), UK (23), Australia (6), Indonesia (6), Cameroon (4), US (3), and Kenya (2). We also received one entry from each of these countries: Botswana, Brazil, Canada, Republic of the Congo, Ghana, Malawi, Malaysia, Netherlands, Sri Lanka, and Uganda.

Judges from a range of countries and diverse professional backgrounds participated, including practising artists and professionals working in healthcare, charity, and sustainability sectors. An evaluation framework to adjudicate the work considered how each piece responded to the competition theme, the creativity and craft involved, and the impact on the audience.

The work of the competition winner and three runners-up are shown below, together with their reflections.

## Winner: Polluted Playground by Elen Evans Studying Medicine at Cardiff University, United Kingdom


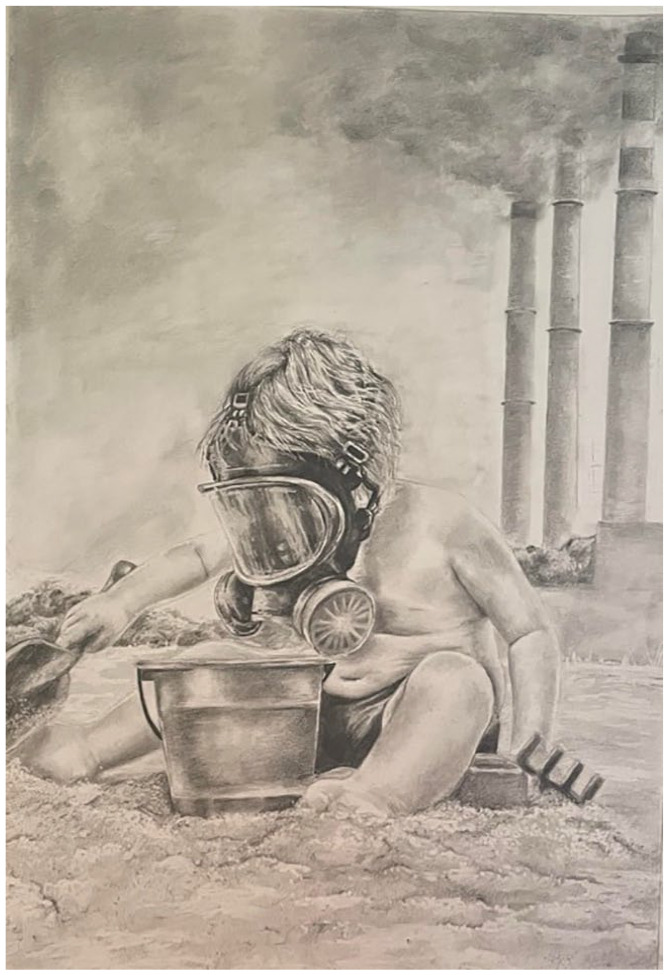

I created this image to cast light on environmental and societal threats posed by climate change.

From the depletion of biodiversity and air pollution to the rise in environmental disasters, the consequences loom large. Future generations will shoulder the weight of health crises, resource shortages, and a world marred by inequality. These burdens will fall most heavily on those from disadvantaged socioeconomic backgrounds. The image of the child playing in the sand reminds us that those yet unborn remain both innocent and susceptible to the environmental repercussions of today’s actions. It underscores our responsibility to prioritise sustainable consumption and production, careful stewardship of natural resources, and pursuit of harmony with the environment.

As a future physician, I will encounter patients affected by changing disease patterns and be constrained in my capacity to deliver effective care. Restoring planetary health is not merely a matter of urgency, it is integral to the wellbeing and longevity of humans.

## Runner-Up: We are Born into Reciprocity by Jamie Thompson Studying Medical Doctorate (MD) at Northern Ontario School of Medicine University, Canada


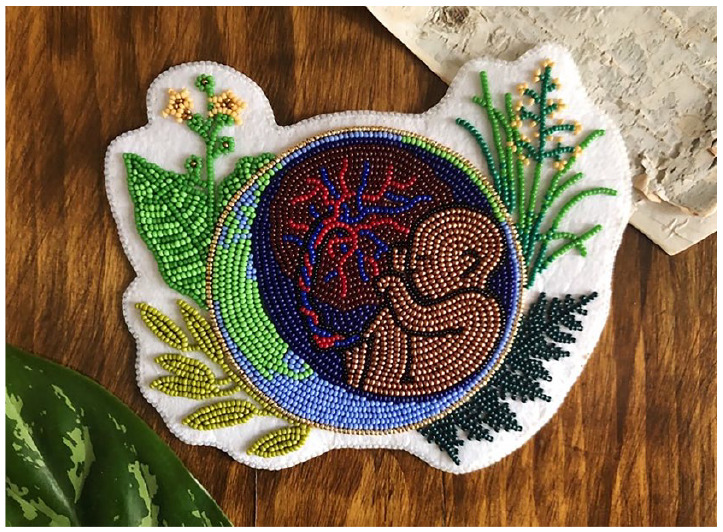

This piece of Métis beadwork represents Indigenous knowledges on environmentalism, sustainability, and global health.

I am a Métis woman and medical student with a passion for preserving Indigenous knowledges and women’s and LGBTQIA+ health. This piece combines my ideas about Indigenous sustainability and reciprocity through the depiction of the foetus and placenta within the ‘womb’ of the earth.

Métis beadwork is a traditional art medium, connecting concepts of matriarchy, land-based practices, and sustainability. The piece depicts the four traditional plant medicines – tobacco, sweetgrass, cedar, and sage – exemplifying how Indigenous knowledges and ways of being promote life on the land, responsible consumption, food security, and sustainable communities.

I recognise my responsibilities to my communities on local and planetary scales. My two worldviews – Western and Traditional – are essential for a sustainable future and fuel my passion for health equity in our changing climate.

## Runner-Up: A Path by Fadhilah Ramadhina Studying Nursing at Universitas Indonesia, Indonesia


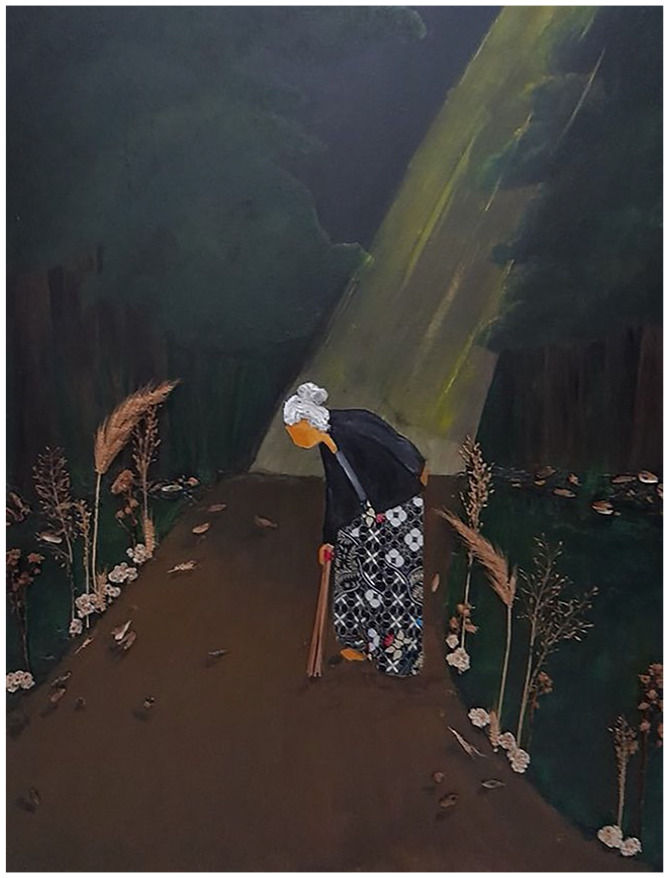

‘aku suka nyapu, biar mati nanti jalannya gampang’ (I like to sweep, so that when I die, my path to the afterlife will be easier).

My painting was influenced by the words of an 80-year-old woman from Yogyakarta, Indonesia. Her statement reminds us that every action shapes not only our present but also our afterlife. By maintaining a clean environment, we contribute to both the wellbeing of our planet and our own health. The act of sweeping embodies physical activity, promoting a healthy lifestyle even in its simplest form. ‘Healthy Inside, Clean Outside’ is the value I bring to this painting – a reminder of the symbiotic relationship between our inner and outer worlds.

## Runner Up: Drinking Water for all by Jonas Nzanzu Studying Biomedical Sciences at Université Simon Kimbangu, Democratic Republic of the Congo

Click here to watch video


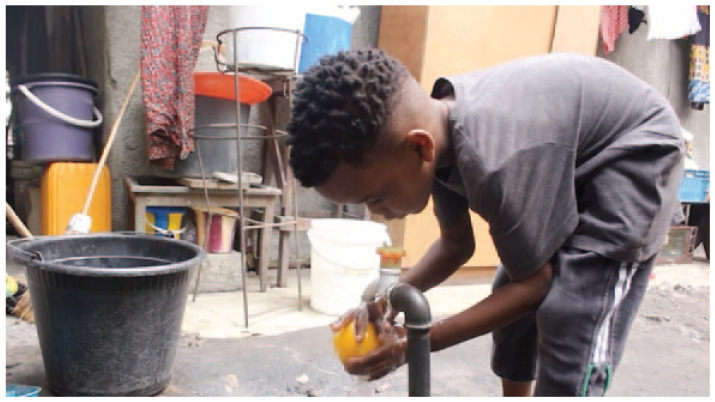

We face a great enemy to health which is water pollution. Children are susceptible to infections that are caused by not having access to safe water to drink and with which to wash their hands. It is essential that people have access to safe drinking water and what could be better than to make this possible?

As a future health professional, I recognise my responsibility to strive to ensure people have safe drinking water close to their homes which can help to ensure good health for all. Through this film I hope that my voice will be heard.

## Discussion

The Global Creative Competition created a unique opportunity for healthcare students from around the world to reflect on the wellbeing of people and the planet. The artworks provided a vehicle through which students could express their views on sustainability and supported knowledge conversations between students studying across health profession disciplines, in both low- and high-income countries. Many entrants expressed an urgent need to change the way we use our earth’s resources and recognised that consequences of the climate crisis, biodiversity loss, resource consumption, hunger, poor sanitisation, and pollution affect people and communities harshly and inequitably. Student submissions also understood the importance of Indigenous knowledges and how such ways of knowing may help shape a more sustainable future. For those adjudicating submissions, it has been inspiring to see our future healthcare workforce’s passion and commitment to ensuring transformational change in our global health systems.
